# RNA-Seq Analysis of Diverse Rice Genotypes to Identify the Genes Controlling Coleoptile Growth during Submerged Germination

**DOI:** 10.3389/fpls.2017.00762

**Published:** 2017-05-15

**Authors:** Sheng-Kai Hsu, Chih-Wei Tung

**Affiliations:** Department of Agronomy, National Taiwan UniversityTaipei, Taiwan

**Keywords:** RNA-Seq, coleoptile, anaerobic germination, rice, diversity, transcriptome

## Abstract

The rate of coleoptile elongation varies between different rice varieties that are grown under water during the germination stage. Compared to sensitive varieties, submergence-tolerant rice exhibits substantial coleoptile elongation in order to uptake oxygen (O_2_) from the surface and thus have a better chance to survive water stress. We conducted RNA-seq analysis in order to investigate 7-day-old shoot transcriptome dynamics in six rice genotypes that exhibit different coleoptile elongation rates under water. This enabled us to identify the genes involved in photosynthesis, lipid metabolism, glycolysis, anaerobic fermentation, hormone synthesis, cell wall growth and elongation, and to demonstrate that these genes are differentially regulated within, and between, genotypes. Further, in addition to determining how allelic variation affects anaerobic germination, we compared the expression patterns and genomic sequences of six genotypes; this enabled us to discover that some genes carry small-to-large deletions in the coding region of sensitive varieties. These structural variations may explain the absence of transcripts in the dataset, as well as the failure of sensitive variety to respond to submergence. On the basis of these results, we hypothesize that transcriptional regulation enhances coleoptile elongation. Although this is an area for future research, the outcome of this study is expected to facilitate rice breeding for direct-seeding.

## Introduction

Elongation of the coleoptile is considered to be an “escape” strategy used by young rice seedlings to tolerate submergence during the germination phase. As a result, a large amount of basic research has been focused on investigating how environmental conditions, cultivar genotypes, seedling physiological characteristics, and seed vigor affect the germination and development of healthy seedlings under hypoxia (Atwell et al., 1982; Yamauchi et al., 1993, 1994; Yamauchi and Chuong, 1995; Yamauchi and Biswas, 1997; Ismail et al., 2009). The results of these studies clearly show that coleoptiles of tolerant cultivars grow faster and longer under submergence, and that this morphological adaptation enables them to reach surface O_2_ faster, allowing diffusion through this structure to other organs including the primary leaf and root to support seedling growth. Indeed, because this so-called “snorkel effect” plays an important role in the early stages of crop establishment when soil is flooded, the development of rice varieties with enhanced capabilities for anaerobic germination will benefit farmers who apply a direct seeding system.

Unlike wheat and most cereal crops, rice is well-known for its capacity to anaerobically mobilize the energy reservoir in the endosperm to support embryonic tissue growth. Previous biochemical and enzymatic experiments have elucidated the mechanisms of starch breakdown and the induction of amylase during low-O_2_ germination in rice seeds (Guglielminetti et al., 1995a,b; Perata et al., 1997; Ismail et al., 2009). These studies have demonstrated a positive correlation between coleoptile length and total amylolytic activities, including α-amylase and the sucrose content of embryos under anaerobic conditions (Pompeiano et al., 2013). Data therefore indicate that the energy supplying coleoptile growth comes from the seed. An elevated level of ethanol production in fast-growing coleoptiles suggests that energy generated from fermentative metabolism supports anoxic growth (Setter et al., 1994; Gibbs et al., 2000; Magneschi et al., 2009), while recent studies have also claimed that maintaining a high rate of energy production as well as a flux between glycolytic and fermentation pathways is crucial for anaerobic tolerance (Edwards et al., 2012; Atwell et al., 2015).

A number of genome-wide transcriptome analyses have been undertaken to investigate the gene expression profiles of rice coleoptiles under hypoxic and anoxic conditions. These experiments were performed at various O_2_ levels, and used expression microarrays to detect the transcription profiles of single rice varieties. The results of these studies revealed that a number of common molecular mechanisms are involved in coleoptile growth, including carbohydrate metabolism, fermentation, hormone induction, cell division, and expansion (Lasanthi-Kudahettige et al., 2007; Huang et al., 2009; Shingaki-Wells et al., 2011; Narsai et al., 2015). Another recent study analyzed gene expression in the tips and basal segments of O_2_-deprived rice coleoptiles, identified the presence of region-specific gene induction, and provided a detailed picture of differential metabolic activities along the length of the coleoptile under normoxic (air), hypoxic (3% O_2_), and anoxic conditions (Narsai et al., 2015).

Rice scientists have carried out a series of genetic mapping analyses using biparental mapping populations and diverse accessions to identify the quantitative trait loci (QTL) associated with anaerobic germination and early seedling growth under submergence (Jiang et al., 2006; Angaji et al., 2010; Septiningsih et al., 2013; Baltazar et al., 2014; Hsu and Tung, 2015). Once detected, these QTLs have been targeted for molecular cloning and marker-assisted breeding (Miro and Ismail, 2013). The first natural variant in QTL *qAG-9-2* to enhance anaerobic germination was recently fine-mapped to *OsTPP7*, a gene that encodes a trehalose-6-phosphate phosphatase (Kretzschmar et al., 2015). Functional characterization of *OsTPP7* suggests involvement in the enhancement of starch mobilization to drive embryo germination and coleoptile elongation. However, although these genetic studies have provided direct evidence that natural variation determines the level of tolerance to flooding at the germination stage in rice, the molecular basis of phenotypic variation remains unknown.

In this paper, we report the results of a comparative RNA-seq analysis using 7-day-old aboveground shoot tissue from six rice genotypes that have differing coleoptile growth when submerged. The aim of this investigation was to identify differentially-expressed transcripts, as well as the molecular mechanisms that contribute to coleoptile growth. The high-resolution capabilities of RNA-seq analysis, combined with the investigation of diverse rice genotypes, allows us to address three questions. First, what is the response of a generic seedling coleoptile to hypoxia? Second, are our transcriptome sequencing results comparable to other expression microarray studies? Third, which genotype-specific expressions are enriched in tolerant varieties and reduced in sensitive ones? We anticipate that the results of this study will not only increase our knowledge of the molecular basis of anaerobic germination but will also facilitate the breeding of tolerant rice varieties for use in cultivation via direct seeding.

## Materials and methods

### Plant material

Six rice genotypes were used in this study: (1) The *japonica* variety Nipponbare; (2) The *indica* variety IR64; (3) Two recombinant inbred lines (RILs; F291 and F274-2a) derived from a cross between Nipponbare and IR64, and; (4) Two accessions that originate from southeast Asia: 8391 from Laos (IRGC 94599) and 8753 from Indonesia (IRGC 54313).

All of the seeds used in this study were freshly harvested and stored at 4°C prior to experiments; sterilized seeds were germinated in capped glass tubes in a growth chamber at 25°C for 7 days under a photoperiod comprising a cycle of 16 h of light (150 μmole m^−2^ s^−1^) and 8 h of dark, designated the “control” condition. For “submergence” treatments, sterilized seeds were germinated in 5 cm of water for 7 days.

Coleoptile length was measured using an ordinary ruler; however, considering that coleoptiles in the control group grown in air were barely elongated, the entire aboveground shoot (i.e., tissue including the coleoptile and primary leaf) of 7-day-old seedlings that showed a consistent response to both air and submergence was harvested and stored at −80°C. Three independent experiments including 15 plants were performed for each accession, and the plant tissues from three independent biological replicates were pooled for RNA isolation.

### Extraction and RNA-Seq library construction and sequencing

Total RNA was extracted using TRI Reagent® (Invitrogen, MA, USA) and a Direct-zol™ RNA MiniPrep Kit (ZymoResearch, CA, USA). RNA samples with an RQI (RNA quality indicator) greater than 7.5 were used for library preparation. Poly-A RNA containing mRNA was purified using poly-T oligo-attached magnetic beads and fragmented, and complementary DNA (cDNA) was synthesized using random hexamer primers, followed by purification, end-repairing, poly-A tailing, and adaptor ligation.

Twelve cDNA libraries comprising unique barcodes were pooled and sequenced in one run using an Illumina HiSeq™ 2500 to generate single-end reads, each 100 base pairs (bp) in length.

### RNA-Seq data analysis

Raw sequenced short reads were aligned to the rice reference genome (MSU7.0) using the CLC Genomics Workbench 6.5 software (CLCbio-Qiagen, Aarhus, Denmark). Expression levels of each gene were quantified by normalizing total exon read counts with effective library size, and tests for pairwise differential expression were performed using the R software package DESeq in Bioconductor (Anders and Huber, 2010). Genes with *P*-values less than 0.05 were considered differentially expressed (DE). These were then used as the basis for biological pathway annotation in the MapMan software (ver. 3.5.1R2) (Usadel et al., 2005). Osa_MSU_v7 mapping files were downloaded from the MapManStore server (http://mapman.gabipd.org/web/guest/mapmanstore).

Applying multi-factorial linear modeling, we were then able to test three null hypotheses of effects on gene expression: (1) whether the expression of each gene was significantly regulated by submergence treatment; (2) whether gene expression was affected by genotype, and; (3) whether gene expression was affected by submergence in a genotype-dependent manner. We therefore fitted our experimental data into four different linear models: (1) FM_trt_: *Y* = τ + ε; (2) FM_geno_: *Y* = τ + ε; (3) FM_add_: *Y* = τ + γ + ε, and; (4) FM_full_: *Y* = τ + γ + τ:γ + ε. In each of these formulae, *Y* is the expression value of each gene, τ is the effect of submergence treatment, *Y* is the effect of different genotypes, and ε is random error. Comparing FM_geno_ and FM_trt_ to FM_add_ separately, we then tested whether the expression of each gene was regulated by submergence, and whether there was a significant genotypic effect. Comparisons of FM_full_ and FM_add_ allowed us to test whether gene expression was affected by submergence in a genotype-dependent manner.

In all cases, expression values for genes were standardized using the expression $z=\left(x-\overset{-}{x}\right)/s_{x}$ for cross-genotype comparisons.

### Quantitative real-time qRT-PCR

Total RNA from 7-day-old aboveground shoot tissues was extracted using TRI Reagent® (Invitrogen, MA, USA) with a Direct-zol™ RNA MiniPrep Kit (ZymoResearch, CA, USA). We included three independent biological replicates in this experiment, and designed gene-specific primers using the NCBI primer BLAST. The complete primer sets used in this study are listed in Table S1, and quantitative RT-PCR was performed using the Rotor-Gene® SYBR® Green RT-PCR kit (Qiagen, Hilden, Germany), following the manufacturer's protocol for one-step RT-PCR. The PCR conditions used in this study encompassed 10 min at 55°C for reverse transcription, 5 min of pre-denaturation at 95°C, 40 cycles of 5 s at 95°C and 10 s at 60°C, followed by melting curve generation. We selected the OsACT1 gene (forward primer: 5′-ATGAAGATCAAGGTGGTCGC-3′; reverse primer: 5′-GTACTCAGCCTTGGCAATCC-3′) as the internal reference for relative quantification, and used the delta-delta C_T_ method to calculate relative expression level fold-changes between submerged and control samples (Pfaffl, 2001).

Log_2_-transformed relative expression levels of different samples were used to evaluate correlation in expression patterns determined by RNA-Seq and qRT-PCR for each selected gene.

### Gene ontology (GO) enrichment analysis

We performed GO enrichment analysis on DE genes using the web-based toolkit “AgriGO” (Du et al., 2010). We used Fisher's exact test and the Benjamini-Hochberg's false discovery rate (FDR) adjustment to control for multiple comparisons (Benjamini and Hochberg, 1995).

### Data availability

All the sequence data generated in this research was deposited in the Experiment ArrayExpress database (https://www.ebi.ac.uk/arrayexpress/) at EMBL-EBI (European Molecular Biology Laboratory-European Bioinformatics Institute) under accession number: E-MTAB-3834.

## Results

### Anaerobic germination and submerged seedling growth

**Figure 1:**
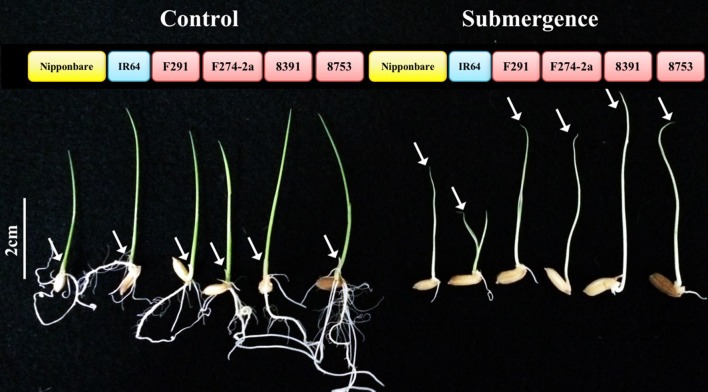
**Seedling growth of six rice genotypes under control and submerged conditions**. Rice seedlings of six genotypes after 7 days under control (air) and submerged (5 cm of water) conditions. The coleoptiles of each genotype are marked with an arrow. Genotype ID was labeled in color based on their submergence tolerance. Blue indicates sensitive genotype, yellow represents moderate tolerance and red indicates extreme tolerance genotypes.

We examined the length of 7-day-old rice seedling coleoptiles across the six rice genotypes (i.e., Nipponbare, IR64, F291, F274-2a, 8391, and 8753) either under normal conditions or submerged (Figure 1, Table 1). The results of Fisher's least significant difference (LSD) tests demonstrated significant variation in coleoptile length when submerged seedlings of different genotypes were compared. Although variation in coleoptile growth between the rice genotypes was also observed under normal conditions, this was not as significant as in submerged examples. Thus, we used coleoptile length differences between submerged and control samples to evaluate the ability of seedlings to tolerate anaerobic stress induced by waterlogging. Results show that the anaerobic response of Nipponbare genotype seedlings was significantly better than the response of the IR64 genotype, and that two Nipponbare/IR64-derived RILs, F291 and F274-2a, both outperformed the former. We have previously confirmed the presence of this significant transgressive variation in an earlier mapping study (Hsu and Tung, 2015), suggesting that epistatic interactions between parental genomes could contribute to anaerobic tolerance. We have also previously identified two extremely tolerant rice landrace genotypes, 8391 and 8753; under submergence the coleoptile length in both these landraces was twice the length of that seen in genotype IR64 seedlings. According to their coleoptile elongation ability, we designated IR64 as sensitive genotype, Nipponbare and others are moderate and extreme tolerant genotypes (Figure 1).

**Table 1:** **Coleoptile lengths of the six rice genotypes analyzed in this study**.

**Genotype**	**Coleoptile length 7 d after germination**		
	**Control (C)**	**Submerged (T)**	**Index (T-C)**
Nipponbare	0.65 ± 0.023^ab^	3.01 ± 0.056^d^	2.36 ± 0.066^d^
IR64	0.47 ± 0.016^c^	2.17 ± 0.160^e^	1.71 ± 0.155^e^
F291	0.42 ± 0.030^c^	3.27 ± 0.115^cd^	2.85 ± 0.113^c^
F274-2a	0.58 ± 0.014^b^	3.61 ± 0.117^c^	3.03 ± 0.130^c^
8391	0.62 ± 0.024^ab^	4.72 ± 0.142^a^	4.10 ± 0.156^a^
8753	0.69 ± 0.034^a^	4.20 ± 0.200^b^	3.51 ± 0.213^b^

*Coleoptile length was measured for each rice genotype under control and submerged conditions and the anaerobic response index was calculated. Length is mean ± standard error (n = 4). One-way ANOVA and Fisher's LSD tests were performed for each trait (each column); numbers followed by different letters are statistically significant following Benjamini-Hochberg's FDR adjustment*.

### RNA-Seq analysis confirms a generic transcriptomic response to submergence, irrespective of genetic background

We constructed 12 cDNA libraries using total RNA purified from the tissue of six genotypes of 7-day-old control (i.e., germinated in air) and submerged aboveground seedlings tissue. We generated a total of 403.65 million short reads (100 bp) from 12 barcoded cDNA libraries in one run for this study using a HiSeq 2500 machine. The detailed sequencing statistics for these cDNA libraries are presented in Table S2, and quality-trimmed reads were aligned to the rice reference genome (i.e., Nipponbare, MSU7.0 annotation). More than 90% of these reads were mapped, and between 87 and 90% were uniquely aligned (Table S3). Total counts of exon-aligned reads were normalized on the basis of effective library size for each sample, using the DESeq software (Anders and Huber, 2010), and the expression signal of each gene was calculated (see above). To further validate transcription expression generated by RNA-seq and DESeq, we selected eight genes based on their read counts and examined their expression levels using RT-PCR. The results of this analysis demonstrated that both quantification platforms generated similar patterns; Pearson's correlation coefficients (*r*) for these eight genes range between 0.63 and 0.96, with an average of 0.79 (Figure S1, Table S4).

**Figure 2:**
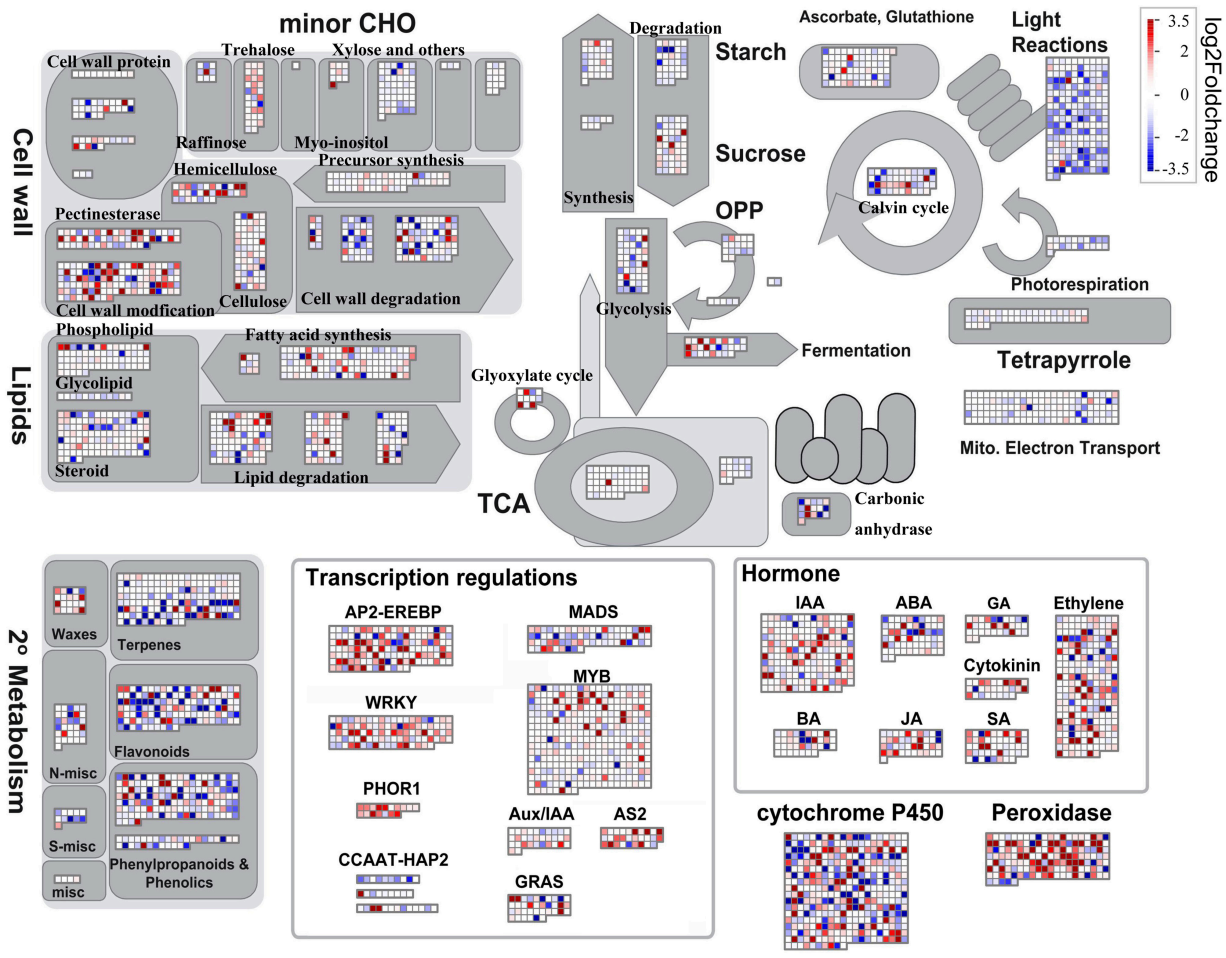
**Biological pathways responded to submergence through transcriptional regulation**. Overview of MapMan biological pathways to illustrate differentially expressed transcripts between control and submerged tissues. The log_2_ fold change color scale ranges from −3.5 to 3.5, with red representing higher gene expression in submerged coleoptiles compared to control shoot tissues, and dark blue representing higher gene expression in control samples compared to submerged coleoptiles. The complete set of genes and calculated ratios is presented in Table S13.

In order to overview the genome-wide transcriptomic changes between control and submerged treatment samples irrespective of their genotypic identities, we averaged the expression signal of each gene across the six genotypes in the control and treatment groups, respectively, and then visualized overall transcriptomic change using the MapMan software (Usadel et al., 2005) to identify metabolic pathways and biological processes regulated under submergence. The results of these comparisons show that genes associated with cell wall modification, fermentation, transcription regulation, and hormone biosynthesis were all up-regulated in the submerged group, while those involved in cell wall degradation, mitochondrial electron transport, photosynthesis, and secondary metabolism were mostly down-regulated (Figure 2).

A submerged rice shoot is mainly composed of elongated coleoptile (Figure 1), a specialized organ that does not produce chlorophyll during anaerobic germination. Our results confirm the expression of photosynthetic genes in control green shoots, while photosynthetic activity was reduced in the submerged coleoptiles (the “light reactions” illustrated in Figure 2). Previous results have shown that the coleoptile is the first organ to senesce when rice seedlings are germinated in air (Inada et al., 2000; Kawai and Uchimiya, 2000). Our results further demonstrate that submergence delays the processes of senescence and cell death, as a significant number of genes were actively induced when seedlings were germinated under water. These genes triggered a series of downstream transcriptional regulators that promote adequate metabolic and morphological adjustments to cope with submergence stress.

### Gene expression in specific genetic backgrounds regulates rapid coleoptile elongation

To further examine whether the gene expression was affected by (1) submergence treatment only, (2) the genotype only, or (3) both genotype and submergence, we performed multi-factor linear model testing on our whole transcriptome dataset using DESeq. Applying a *P*-value of less than 0.05 as the cutoff, our results show that 3,597 genes were significantly affected by submergence regardless of their genetic backgrounds (i.e., the “treatment effect” test in Table S5), while 5,100 genes were differentially expressed across six genotypes irrespective of submergence (i.e., the “genotype effect” test in Table S5), and the expression of 471 genes was affected by submergence in a genotype-dependent manner (i.e., the “interaction” test in Table S5). These results therefore provide the first confirmation that gene expression under submergence can be regulated differently depending on genetic background; in other words, variation of coleoptile elongation in diverse rice accessions could potentially be determined by genotype-specific gene expression. We performed GO enrichment analysis separately for up-regulated and down-regulated gene sets of the 3,597 submergence-responsive genes from linear modeling by the AgriGO software (Du et al., 2010). The results of this analysis reveal a similar pattern to MapMan visualization (Figure 2)—that genes responsible for oxidative stress responses, transcriptional regulation, post-translational regulation, and cell wall organization are specifically enriched in the up-regulated gene set, while those responsible for carbon fixation, carboxylic acid metabolism, and a number of other macromolecular metabolic processes are all down-regulated (Table S6).

In order to investigate in detail the distribution of DE genes in our six diverse transcriptomic profiles, we next analyzed gene expression in submerged and control samples genotype-by-genotype independently using negative binomial testing in DESeq, and then applied a *P*-value less than 0.05 as a cutoff to generate a DE gene list for each genotype. Using this approach, we identified 701 and 808 DE genes in the two extremely tolerant landrace genotypes 8391 and 8753, total 692 genes in the moderately tolerant cultivar Nipponbare genotype, and just 193 in the sensitive cultivar genotype IR64 (Figure S2). According to the MA plots (Figure S3), all DE genes showed significant differential expression (log_2_ Fold Change > 2 or < −2) and strong expression (mean expression > 100); this suggests that a *P*-value < 0.05 is a reasonable threshold in this study to identify differential expressed genes. In addition, 83 DE genes (i.e., 68 up-regulated and 15 down-regulated) were commonly present in four rice varieties (Nipponbare, IR64, 8391, 8753), while a number of genotype-specific DE genes can also be observed (Figure S2, Table S7). Our findings suggest that rice coleoptile elongation when submerged at the germination stage has been fine-tuned based on differences in gene expression pattern in different genetic backgrounds.

In the two RILs derived from the Nipponbare and IR64 genotypes, we identified 527 and 929 DE genes in F291 and F274-2a, respectively, while 93 genes were uniquely expressed in recombinants. These data imply that unique expression of these genes contributes to the transgressive phenotype and leads to rapid coleoptile growth (Figure S2, Table S7).

**Figure 3:**
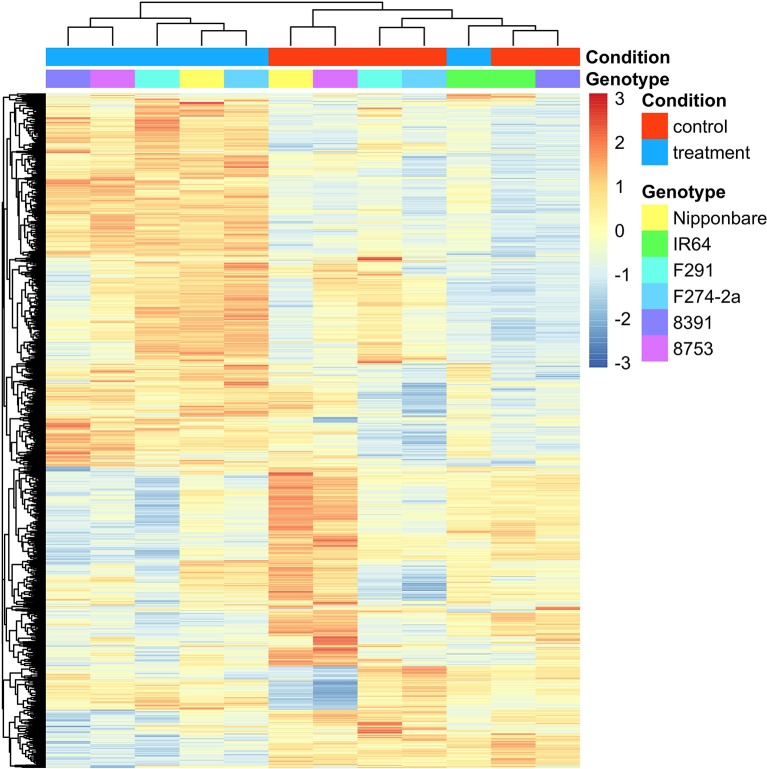
**Expression patterns of the 2,026 DE genes in the six rice genotypes**. This figure shows the normalized expression value of DE genes in each sample along a scale from −3 to 3, with the red color representing higher expression and the blue color representing lower expression. Clustering was performed among both selected genes and across different samples, while sample identity is annotated at the top of each column.

As revealed by MapMan analysis (Figure S4), because transcriptomic activity was significantly reduced in the sensitive IR64 cultivar comparing to other tolerant genotypes, it is possible that poor coleoptile elongation in this case could be attributed to weak transcriptomic changes. Unlike the other five genotypes, IR64 exhibited poor transcriptomic responses under submergence, both in terms of the number of responsive genes and the level of responsiveness (Figure 3 and Figure S5). Thus, based on the results of pairwise DE analyses, we identified sets of genes of interest according to the following criteria: (1) Genes that were responsible for fundamental submergence responses irrespective of genotype; (2) Genes that were differentially expressed in the five tolerant lines but that responded poorly in the sensitive IR64 genotype; (3) Genes that promoted coleoptile elongation in the four rapidly-growing genotypes, and; (4) Genes that exhibited an absence of transcripts in the sensitive IR64 genotype.

#### Genes that consistently exhibit significant differential regulation in all six genotypes

**Figure 4:**
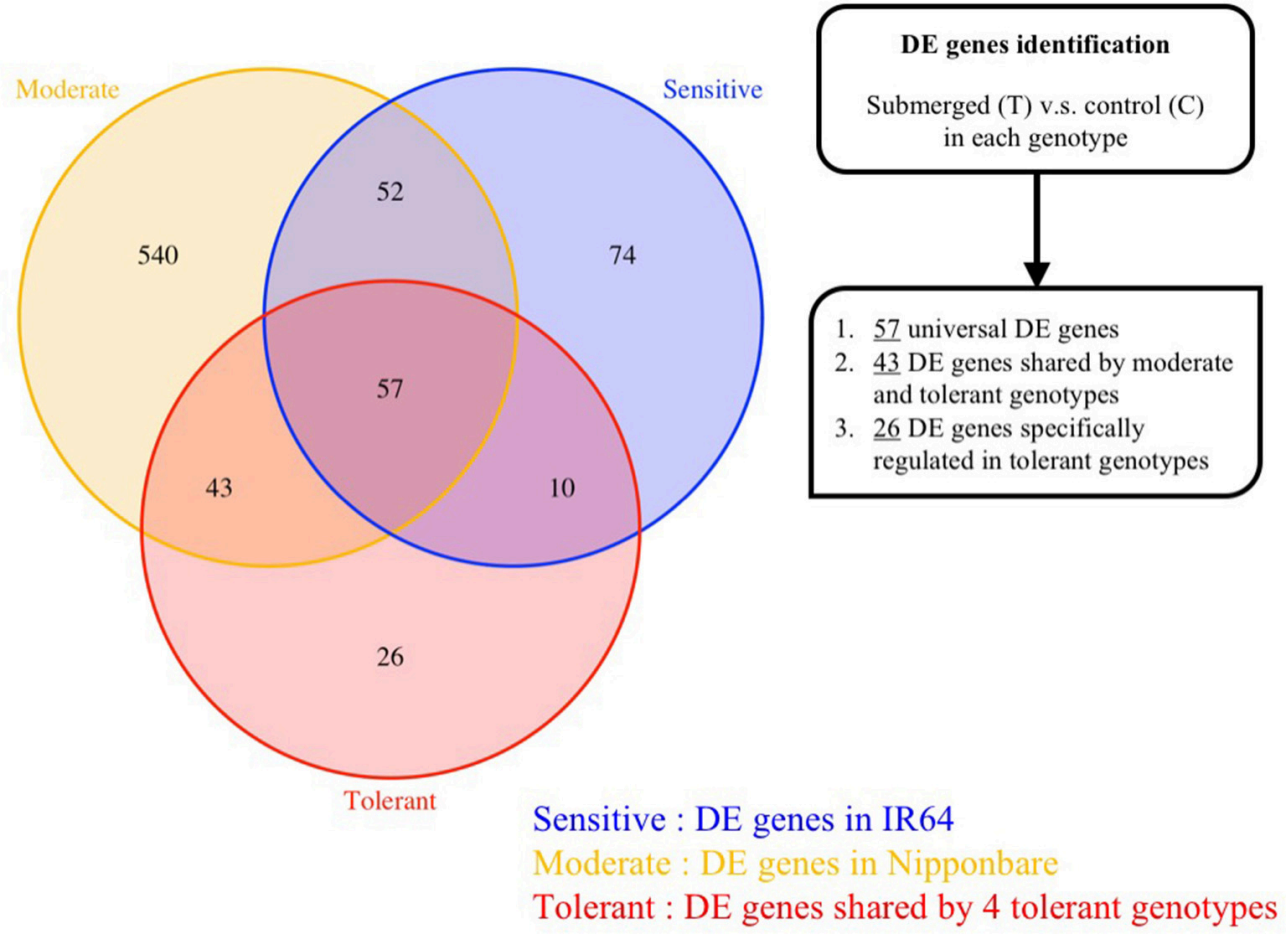
**DE genes identified by comparing expression between control and submerged shoots in each genotype**. Three-way Venn diagram to illustrate the number of DE genes that are either unique to, or shared between genotypes. The colored lines correspond to tolerance level described in Figure 1. The number in each block indicates the number of DE genes in each group.

Among the overall set of 2,026 submergence-responsive DE genes from six genotypes, 57 were conservatively regulated in all genotypes (Figure 4, Table 2, Table S7) and included genes encoding the important fermentative enzymes, pyruvate decarboxylase 1 (*PDC1, LOC_Os05g39310*), which channels pyruvate into the alcohol fermentation pathway (Hossain et al., 1996; Gibbs et al., 2000) and alcohol dehydrogenase 2 (*ADH2, LOC_Os11g10510*), which converts acetaldehye to ethanol (Perata and Alpi, 1991), together with the gene encoding vacuolar proton phosphatase (H^+^-PPase, a pyrophosphate-related active proton transporter that maintains cytosolic pH homeostasis—*LOC_Os02g55890*) (Liu et al., 2009), as well as those encoding proteins related to cell wall or membrane structure (*LOC_Os01g67030, LOC_Os10g40510*, and *LOC_Os08g40690*). Although these genes have been previously reported to be involved in fundamental submergence response mechanisms (Lasanthi-Kudahettige et al., 2007; Narsai et al., 2009), they were consistently regulated in all six genotypes, and the strength of their response to submergence (i.e., fold change of expression) varies. Thus, based on these initial results, we suggest that stronger induction, or suppression, of these fundamentally responsive genes can enhance the submergence tolerance of tolerant genotypes.

**Table 2:** **Differentially expressed gene responses to submergence during germination in contrasting rice genotypes**.

**Gene id**	**Log_2_ fold change (treatment/control)**						
	**IR64**	**Nipponbare**	**F291**	**F274-2a**	**8391**	**8753**	
**57 DE GENES SIGNIFICANTLY DETECTED IN SIX GENOTYPES (*P* < 0.05)**							
LOC_Os01g09030	2.54	4.18	2.46	3.73	3.88	5.62	Putative uncharacterized protein
LOC_Os01g09540	−2.66	−6.09	−4.96	−2.79	−4.85	−6.11	Putative acid phosphatase
LOC_Os01g19820	3.46	3.75	3.65	5.78	3.55	3.22	Putative ER6 protein
LOC_Os01g21120	3.11	6.04	2.97	5.58	5.27	3.96	Putative ethylene response factor 2
LOC_Os01g22249	5.33	3.97	7.11	8.15	8.19	8.07	Class III peroxidase 13
LOC_Os01g58290	4.34	3.33	3.35	4.87	5.06	5.89	Uncharacterized protein
LOC_Os01g65830	4.18	5.73	3.39	4.89	6.36	6.60	Acyl-[acyl-carrier-protein] desaturase 1
LOC_Os01g67010	5.56	8.20	3.84	8.48	9.05	7.75	Uncharacterized protein
LOC_Os01g67030	3.81	10.53	5.75	11.27	9.42	7.96	Membrane protein-like protein
LOC_Os01g68300	−2.77	−3.83	−6.56	−2.57	−7.91	−7.21	Uncharacterized protein
LOC_Os01g68720	6.69	4.06	5.81	7.24	5.93	5.78	Uncharacterized protein
LOC_Os01g74410	3.16	3.15	4.44	6.65	5.44	4.78	Putative Myb factor protein
LOC_Os02g39620	Inf	7.21	6.54	8.94	9.92	Inf	Putative stress-inducible protein
LOC_Os02g55890	4.32	7.11	6.80	6.13	6.46	6.91	Vacuolar proton pyrophosphatase
LOC_Os03g01320	2.31	2.51	4.08	5.39	5.66	3.52	Retrotransposon protein, Ty1-copia subclass
LOC_Os03g03034	4.10	3.87	2.75	4.06	2.94	4.64	Oxidoreductase, 2OG-Fe oxygenase family protein
LOC_Os03g08460	8.15	4.30	6.79	8.70	8.23	6.22	AP2 domain containing protein
LOC_Os03g12510	Inf	9.63	5.16	6.83	Inf	5.13	Non-symbiotic hemoglobin 2
LOC_Os03g19270	3.16	5.37	3.13	4.49	4.54	5.47	Universal stress protein family protein
LOC_Os03g19600	5.65	2.86	4.22	6.27	6.84	6.28	Retrotransposon protein, Ty3-gypsy subclass
LOC_Os03g45250	5.62	6.62	4.39	6.16	7.15	7.38	Uncharacterized protein
LOC_Os03g49524	4.16	4.21	3.85	7.01	4.23	5.18	Uncharacterized protein
LOC_Os03g55680	6.54	Inf	6.58	9.34	7.25	9.13	Uncharacterized protein
LOC_Os03g61150	6.67	5.95	3.15	6.04	7.75	7.08	Uncharacterized protein
LOC_Os04g17660	2.48	3.87	5.09	5.58	4.73	6.91	Uncharacterized protein
LOC_Os04g31790	4.12	5.60	3.90	5.51	4.36	4.81	Uncharacterized protein
LOC_Os04g41620	−4.89	−7.91	−Inf	−6.82	−Inf	−8.60	CHIT2—Chitinase family protein precursor
LOC_Os04g56430	6.89	6.49	7.69	7.37	10.54	7.46	Putative receptor-like protein kinase
LOC_Os05g06920	−5.18	−4.22	−5.81	−4.89	−3.80	−6.06	Uncharacterized protein
LOC_Os05g37780	Inf	7.50	4.36	7.66	7.97	8.62	Uncharacterized protein
LOC_Os05g39310	2.68	4.92	4.26	5.00	3.71	6.30	Pyruvate decarboxylase 1
LOC_Os06g04940	Inf	8.72	7.59	7.88	8.34	9.31	Uncharacterized protein
LOC_Os07g24830	9.51	5.73	7.11	9.82	−3.06	6.56	Uncharacterized protein
LOC_Os07g32680	3.37	3.75	4.72	5.17	8.02	6.22	Putative glycine-rich cell wall structural protein
LOC_Os07g32710	4.56	3.61	3.39	6.15	5.71	5.69	Uncharacterized protein
LOC_Os07g41350	Inf	Inf	3.86	7.52	7.86	8.27	Uncharacterized protein
LOC_Os07g44140	3.12	5.04	2.80	4.68	6.17	2.67	Putative cytochrome P450
LOC_Os07g47790	6.76	9.08	6.42	7.46	9.38	8.84	AP2 domain transcription factor EREBP
LOC_Os08g04210	6.98	6.56	8.38	9.12	7.77	9.91	33-kDa secretory protein
LOC_Os08g08100	6.24	6.70	5.30	7.48	8.47	9.19	Uncharacterized protein
LOC_Os08g40690	5.03	6.96	7.69	7.29	9.24	8.83	Chitinase
LOC_Os09g31000	Inf	7.89	8.70	Inf	8.57	8.49	Uncharacterized protein
LOC_Os09g36680	−5.80	−5.86	−4.73	−6.03	−6.70	−11.68	Drought-induced S-like ribonuclease
LOC_Os09g36930	−3.93	−6.17	−5.79	−4.67	−Inf	−5.87	Probable aquaporin PIP2-7
LOC_Os09g38910	4.37	3.97	3.51	4.61	6.01	3.40	Uncharacterized protein
LOC_Os10g30150	Inf	10.47	2.97	6.31	7.68	8.42	Universal stress protein family protein
LOC_Os10g31420	Inf	Inf	9.18	5.69	6.78	6.46	Uncharacterized protein
LOC_Os10g40510	6.66	7.46	7.50	8.71	8.46	8.19	Cortical cell delineating protein
LOC_Os10g40530	3.72	4.03	4.10	6.55	5.20	5.35	Cortical cell delineating protein
LOC_Os11g10510	3.39	8.38	5.29	6.16	6.03	5.90	Alcohol dehydrogenase 2
LOC_Os11g42500	−4.17	−5.49	−6.00	−3.44	−6.08	−6.88	Dirigent-like protein
LOC_Os11g47500	3.87	5.97	7.31	7.28	8.39	7.49	Xylanase inhibitor protein 1
LOC_Os11g47570	4.00	5.34	6.13	7.69	8.36	6.45	Uncharacterized protein
LOC_Os11g47600	5.22	8.20	4.97	8.90	8.52	6.55	Xylanase inhibitor protein 1
LOC_Os12g09540	3.20	4.09	3.99	4.05	4.45	6.30	Phosphoribosylamine-glycine ligase
LOC_Os12g35610	2.83	6.17	3.97	5.77	5.03	6.49	Respiratory burst oxidase
LOC_Os12g38770	4.49	4.34	6.49	5.93	6.65	5.60	Nucleotide pyrophosphatase/phosphodiesterase
**43 DE GENES STRONGLY REGULATED IN FIVE TOLERANT GENOTYPES (*P* < 0.05) BUT LESS SIGNIFICANT IN SENSITIVE IR64 (*P* > 0.05)**							
LOC_Os01g13690	−2.03	−5.05	−4.75	−3.64	−3.56	−5.19	Branched-chain amino acid aminotransferase-like protein
LOC_Os01g45274	−1.04	−3.21	−2.76	−2.35	−2.59	−4.57	Carbonic anhydrase
LOC_Os01g46120	−1.26	−4.39	−4.97	−4.21	−4.94	−5.51	Lipase-like protein
LOC_Os01g70520	−1.70	−6.51	−6.36	−5.38	−Inf	−8.73	Beta-glucosidase 5
LOC_Os02g02400	−1.61	−6.40	−8.44	−5.19	−8.12	−9.24	Catalase isozyme A
LOC_Os02g18650	Inf	7.09	7.53	6.61	6.18	7.30	Pectinesterase
LOC_Os02g20540	−1.60	−5.60	−5.97	−3.18	−7.50	−5.82	Putative fasciclin-like arabinogalactan-protein
LOC_Os02g30080	−1.91	−4.73	−7.27	−4.79	−6.78	−5.46	Cytochrome P450 family protein
LOC_Os02g38920	1.60	3.04	4.35	4.17	4.98	4.35	Glyceraldehyde-3-phosphate dehydrogenase 3, cytosolic
LOC_Os03g13300	1.62	7.58	2.68	4.28	5.95	2.78	Glutamate decarboxylase
LOC_Os03g27190	−1.63	−4.27	−6.02	−6.46	−6.48	−8.55	ICE-like protease p20 domain containing protein
LOC_Os03g31750	2.08	5.43	6.38	5.00	6.24	6.78	Pyruvate, phosphate dikinase
LOC_Os03g45619	−1.95	−9.38	−5.41	−4.31	−6.70	−13.01	Cytochrome P450 family protein
LOC_Os03g57490	1.40	5.28	4.68	5.86	5.35	4.71	Beta-Ig-H3 domain-containing protein
LOC_Os04g25650	1.65	4.50	5.94	4.87	6.20	3.75	Uncharacterized protein
LOC_Os04g54220	4.12	5.94	6.65	8.03	7.53	7.48	Uncharacterized protein
LOC_Os04g56230	−1.21	−6.00	−Inf	−6.43	−5.76	−6.83	Polyprenyl synthetase
LOC_Os05g10650	1.68	5.50	3.96	4.87	5.65	4.21	Uncharacterized protein
LOC_Os05g12400	−2.83	−5.56	−8.49	−3.46	−6.12	−8.47	BURP domain-containing protein 1
LOC_Os05g12630	−3.12	−6.63	−4.22	−3.02	−4.74	−7.14	BURP domain-containing protein 7
LOC_Os05g13970	Inf	6.16	6.59	6.29	9.22	5.65	Uncharacterized protein
LOC_Os06g03520	2.14	6.07	2.90	2.85	8.35	6.83	Uncharacterized protein
LOC_Os06g05020	1.88	8.48	2.84	3.17	8.41	8.75	Putative early nodulin
LOC_Os06g49340	1.89	Inf	4.95	6.46	5.38	9.90	Uncharacterized protein
LOC_Os07g01560	1.71	5.13	3.56	4.56	6.11	3.45	Putative monosaccharide transport protein MST1
LOC_Os07g04990	−2.19	−4.58	−3.97	−3.92	−3.38	−6.39	Aldo/keto reductase family-like protein
LOC_Os07g09630	1.82	5.43	4.12	4.35	3.84	6.40	Uncharacterized protein
LOC_Os07g34570	−1.53	−3.29	−7.22	−4.45	−5.66	−7.52	Thiamine thiazole synthase, chloroplastic
LOC_Os07g37454	2.56	6.46	3.44	5.13	7.48	6.40	Uncharacterized protein
LOC_Os07g46480	−1.67	−4.32	−3.36	−2.77	−4.33	−6.03	Nucleoid DNA-binding-like protein
LOC_Os07g47990	1.32	3.71	5.32	4.68	6.51	3.34	Putative peroxidase
LOC_Os07g49110	−1.26	−4.80	−5.03	−3.86	−5.39	−5.11	Uncharacterized protein
LOC_Os08g25720	0.35	2.58	3.07	3.08	2.45	2.94	Putative pyrophosphate-dependent phosphofructokinase
LOC_Os08g27840	−0.65	−4.07	−4.71	−2.62	−3.74	−4.04	Phosphoenolpyruvate carboxylase
LOC_Os09g16520	1.23	4.29	6.19	5.60	7.09	6.56	Cytochrome b5-like Heme/Steroid binding domain containing protein
LOC_Os09g31040	2.18	5.90	6.71	5.93	7.98	8.21	Uncharacterized protein
LOC_Os10g23180	−2.22	−7.12	−7.18	−6.30	−8.44	−7.37	Cytochrome P450 family protein
LOC_Os10g26700	1.97	Inf	6.49	6.97	5.64	8.27	YGL010w
LOC_Os10g31460	−0.69	5.25	8.41	4.38	10.02	3.95	Uncharacterized protein
LOC_Os11g43860	−0.64	−4.19	−4.17	−7.00	−3.94	−4.48	Magnesium/proton exchanger 1
LOC_Os11g47550	NA	Inf	10.47	7.02	8.27	5.88	Xylanase inhibitor protein 2
LOC_Os11g47560	4.19	Inf	5.90	7.24	Inf	Inf	Xylanase inhibitor protein 2
LOC_Os11g47590	Inf	6.02	6.25	Inf	7.40	7.80	Xylanase inhibitor protein 1

*The original P-value for each gene is listed in Table S7. Gene descriptions were extracted from the Gramene BioMart database and MSU gene annotation*.

#### Genes that exhibit differential regulation in the tolerant lines (*P* < 0.05) but are not significantly regulated in the sensitive IR64 genotype (*P* > 0.05)

Genes that are responsive to submergence in the five tolerant genotypes, but not in the sensitive genotype IR64, are strong candidates for involvement in anaerobic germination. Therefore, we identified 43 genes that were significantly regulated in the five tolerant lines, but not significantly so in the sensitive IR64 genotype (Figure 4, Table 2, Table S7). These results show that genes encoding glycolytic enzymes, such as pyrophosphate-dependent phosphofructokinase (PPi-PFK, *LOC_Os08g25720*), glyceraldehyde-3-phosphate dehydrogenase (G3PDH, *LOC_Os02g38920*), and pyruvate pyrophosphate dikinase (PPDK, *LOC_Os03g31750*), were significantly up-regulated, while significant down-regulation was seen in genes encoding phosphoenolpyruvate carboxylase (PEPC, *LOC_Os08g27840*), cytochrome P450 (*LOC_Os03g45619* and *LOC_Os10g23180*), and catalase (*LOC_Os02g02400*). Of these, PEPC encodes an enzyme that drains PEP from glycolysis via a reaction that converts it into oxaloacetate. The expression patterns of significant DE genes across tolerant genotypes was confirmed under submergence in a previous single genome transcriptome study (Lasanthi-Kudahettige et al., 2007), which implied conservation of a basal anaerobic germination mechanism across tolerant and moderately-tolerant genotypes. In contrast, we suggest that due to the absence of these basal responses, IR64 is not able to maintain normal seedling growth and perform poorly when submerged.

#### Significantly responsive genes in four genotypes with rapidly growing coleoptiles

In addition to the identification of fundamental tolerance-related gene regulation shared by all five tolerant genotypes, DE genes that are exclusively present in the four extremely tolerant lines (F291, F274-2a, 8391, and 8753) may explain the rapid growth of submerged coleoptiles. We identified 26 genes that are specifically regulated in the four highly tolerant genotypes (Figure 4, Table S7), including several that are related to membrane structure and cell walls (*LOC_Os07g35480, LOC_Os10g40430, LOC_Os10g40440*, and *LOC_Os10g40520*). In addition, of the 58 genes that are significantly regulated in the two natural accessions (8391 and 8753), we identified strong up-regulation in several cell wall modification-related genes, including genes encoding a cellulase (*LOC_Os10g22570*), an expansin (*EXP-B6, LOC_Os10g42610*), a xyloglucan endotransglucosylase/hydrolase (*LOC_Os11g33270*), and an AP2-domain that contains ethylene response transcription factor (ERF, *LOC_Os01g04800*). These observations imply that genotypes with rapidly growing coleoptiles share similar mechanisms related to ethylene signaling and cell wall modification which enable faster cell elongation to withstand and escape submergence. At the same time, different genes are activated to trigger tolerance when different genotypes are submerged.

#### Structural variations and their effects on tolerance of anaerobic germination

Because diverse genetic accessions allow us to investigate the effects of structural variation on phenotypes, it is possible to determine whether, in specific cases, gene expression may be absent because of large deletions. Thus, by comparing the transcriptomes of all genotypes under both control and treatment conditions, we identified a set of 109 genes that are expressed at high levels in the five tolerant lines but are barely detectable in IR64 (Table S8). While most of these were annotated as “unknown expressed,” two interesting genes caught our attention; of these, one encodes trehalose-phosphate phosphatase (*OsTPP7, LOC_Os09g20390*), reported to be the causal gene in a series of anaerobic germination QTL mapping studies (Angaji et al., 2010; Kretzschmar et al., 2015), while the other encodes a crucial enzyme for ethylene biosynthesis, 1-aminocyclopropane-1-carboxylate oxidase (*ACC oxidase 1, ACO1, LOC_Os09g27820*). Because fine-scale analysis of *OsTPP7* has demonstrated the presence of a 20.9 Kb truncation in the sensitive genotype IR64 yet failed to promote anaerobic germination (Kretzschmar et al., 2015), it is of interest to further elucidate the expression patterns of *OsTPP7* in our six genotypes. Our results show that while *OsTPP7* was not detected in the IR64 genotype, it was up-regulated in both Nipponbare and the two RIL genotypes, and down-regulated in the two landraces (Table S8). The results of previous transcriptomic studies have also confirmed that some of these genes are responsive to anaerobic stress during early germination. Thus, by investigating the genomic sequences of these 109 genes downloaded from the International Rice Research Institute (IRRI) Rice SNP-Seek Database (http://oryzasnp.org/iric-portal/), we discovered that some carry small-to-large deletions in the IR64 coding region (Table S8), which may explain the absence of transcripts in our IR64 RNA-seq dataset. We therefore hypothesize that some of these genes failed to respond to submergence because of structural variations and that this resulted in slow coleoptile elongation in IR64. The function of these genes in anaerobic germination remains to be investigated.

## Discussion

### The differential modulation of submergence responsive mechanisms leads to contrasting tolerance across diverse genotypes

Several previous studies have investigated the expression of target genes and the biological pathways involved in coleoptile growth during anaerobic germination (Magneschi et al., 2009; Miro and Ismail, 2013; Lee et al., 2014). In this study, we confirm the regulation of previously reported submergence responsive pathways in tolerant genotypes, although some modulation defects were found in the sensitive cultivar IR64, accounting for its poor seedling growth performance when submerged. Additional genes potentially involved in cell wall biosynthesis or modification pathways were strongly regulated in submerged seedlings of highly tolerant genotypes, promoting faster coleoptile elongation to escape submergence.

**Figure 5:**
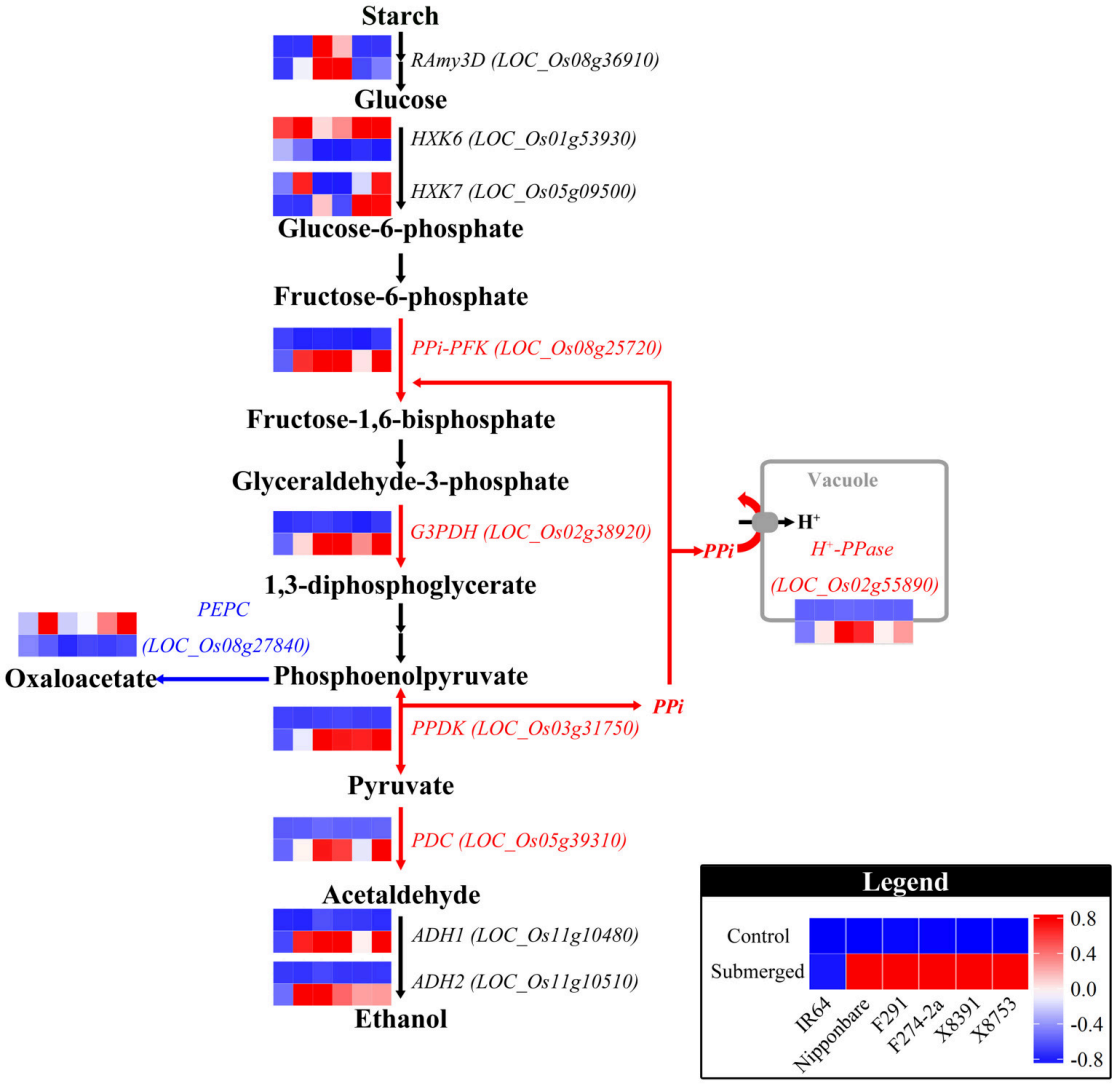
**Genotypic expression of genes involved in starch degradation, glycolysis, and ethanol fermentation**. The pathway shown in this figure was modified from Lasanthi-Kudahettige et al. (2007) with gene expression standardized (see Materials and Methods) to allow for cross-genotype comparisons. The red arrows in this figure highlight significantly induced reactions in the five tolerant genotypes compared to IR64, while the blue arrow denotes increased reduction of PEP carboxylase.

In order to comprehensively discuss the molecular basis of coleoptile growth variation across our six diverse genotypes, we selected three major biological processes that involve large numbers of the candidate genes identified in this study and that have been studied intensively in the context of rice anaerobic germination (Figure 5, Figure S6). We compared and discussed the expression patterns of these target genes across our six genotypes.

### The adjustment of carbohydrate metabolism in response to submergence

Rice seedlings take up water in order to germinate; during the first 48 h of this process, metabolic activity is reactivated and starch in the endosperm is degraded, followed by hydrolysis and glycolysis. The energy generated by these biochemical processes is then used for embryonic tissue growth, and the first organ to protrude is the coleoptile. Elongation of this structure requires energy for cell growth and maintenance; when O_2_ is limited, carbohydrate catabolism shifts to anaerobic respiration via pyruvate metabolism, correlated with an increase in the expression of genes involved in fermentative pathways (Lasanthi-Kudahettige et al., 2007; Narsai et al., 2009).

Building on these previous observations, we investigated the genes involved in starch and sucrose mobilization across our six genotypes, including those encoding α-amylase, sucrose synthase (SuSy), ADP-Glc PPase, UTP-Glc-1-P uridylyltransferase, phosphoglucomutase (PGM), and hexokinase (Table S9). Two of these enzymes in particular are considered essential for carbohydrate catabolism in rice seedling germination, α-amylase for starch degradation and sucrose synthase for sucrose breakdown. Previous work has shown that *RAmy3D* expression can be induced in response to an O_2_ deficit during early germination (Guglielminetti et al., 1995a,b; Perata et al., 1997; Hwang et al., 1998, 1999), and that expression between 24 and 72 h after germination is positively correlated with shoot growth (Ismail et al., 2009). Our data show that of the ten α-amylase encoding genes we analyzed, only *RAmy3D* (*LOC_Os08g36910*) was up-regulated in the five tolerant genotypes and was barely detectable in IR64. At the same time, correlation between the induction level of this gene and coleoptile elongation rate was not linear in our tolerant varieties, an observation that could be explained by our late sample collection time. We observed a similar regulation pattern to that reported in previous work (Magneschi and Perata, 2009) in the case of the Nipponbare genotype; the activity of glycolytic genes, with the exception of those encoding sucrose synthase 1 (*SuSy1*), hexokinase 7 (HXK7), and PGM, were accelerated in the anoxic embryo and coleoptile tissue (Lasanthi-Kudahettige et al., 2007; Narsai et al., 2009). Indeed, as previously described, the genes involved in glycolytic processes, including those encoding G3PDH (*LOC_Os02g38920*) and PPDK (*LOC_Os03g31750*), were all significantly induced, albeit at different levels, in the five tolerant genotypes but not in the sensitive IR64 (Figure 5). This differential regulation could control the rate of glycolysis and influence pyruvate production for fermentative pathways, resulting in variation in coleoptile growth.

Another example of differential modulation of the carbohydrate metabolism pathway is expression of the gene encoding phosphoenolpyruvate carboxylase (PEPC, *LOC_Os08g27840*). Because strong suppression of this gene was detected in tolerant lines and a lower level of reduction was observed in the IR64 genotype (Figure 5), we hypothesize that the presence of PEPC in the submerged IR64 coleoptile might result in insufficient pyruvate for ethanol production. Thus, because the gene encoding PEPC is significantly expressed in aerobic tissue; when it is unrepressed it will convert PEP into oxaloacetate (Figure 5), bypassing alcohol fermentation.

Previous work has also demonstrated that the activities of enzymes involved in fermentative ethanol production, including pyruvate decarboxylase (PDC) and alcohol dehydrogenase (ADH), are significantly higher in seeds germinating under hypoxia in the tolerant “Khaiyan” rice variety than they are in the sensitive “IR42” variety (Ismail et al., 2009). We detected significant induction of genes encoding both PDC (*LOC_Os05g39310*) and ADH (*LOC_Os11g10480* and *LOC_Os11g10510*) in the five tolerant genotypes but not in IR64 (Figure 5), suggesting that ethanol production could be impaired in the sensitive genotype via transcriptional regulation, leading to insufficient energy generation for coleoptile growth.

### The pyrophosphate (PPi)-dependent energy supply pathway under submergence

Several previous studies have suggested that because ATP-consuming processes are not favored by hypoxia, PPi should be considered as an alternative energy donor to potentially maintain cell growth (Gibbs and Greenway, 2003; Magneschi and Perata, 2009; Atwell et al., 2015). Thus, to elucidate the contribution of ATP- and PPi-dependent enzymes in anaerobic germination, we investigated the expression patterns of genes encoding ATP-dependent phosphofructokinases (ATP-PFK), PPi-dependent phosphofructokinases (PPi-PFK or PPi-PFP), vacuolar proton pyrophosphatase (V-PPase), and vacuolar proton ATPase (V-ATPase). Our results show that genes encoding two ATP-PFKs (*OsPFK4* and *OsPFK5*), one PPi-PFK (*OsPFPA3*), and one V-PPase (*OVP3*) were significantly induced in hypoxia samples of our five tolerant genotypes, while the degree of induction in the IR64 genotype was merely detectable (Table S10). The expression levels of PPi-PFK genes in our hypoxia samples were significantly higher when compared to ATP-PFK, which suggests that elongation of the coleoptile may rely heavily on PPi-PFK to phosphorylate fructose-6-phosphate to fructose-1,6-bisphosphate in glycolysis. In addition, the vacuolar proton pyrophosphatase gene family has been shown to actively pump H^+^ from the cytosol into the vacuole to maintain cytosolic pH homeostasis (Maeshima, 2000). In particular, the *OVP3* gene (*LOC_Os02g55890*) has been previously reported as responsive to anoxia (Liu et al., 2009); this gene was detected as differentially regulated across the six genotypes in this study, suggesting an important role in submergence tolerance.

### Genes and mechanisms involved in cell elongation and the ethylene signaling pathway

**Figure 6:**
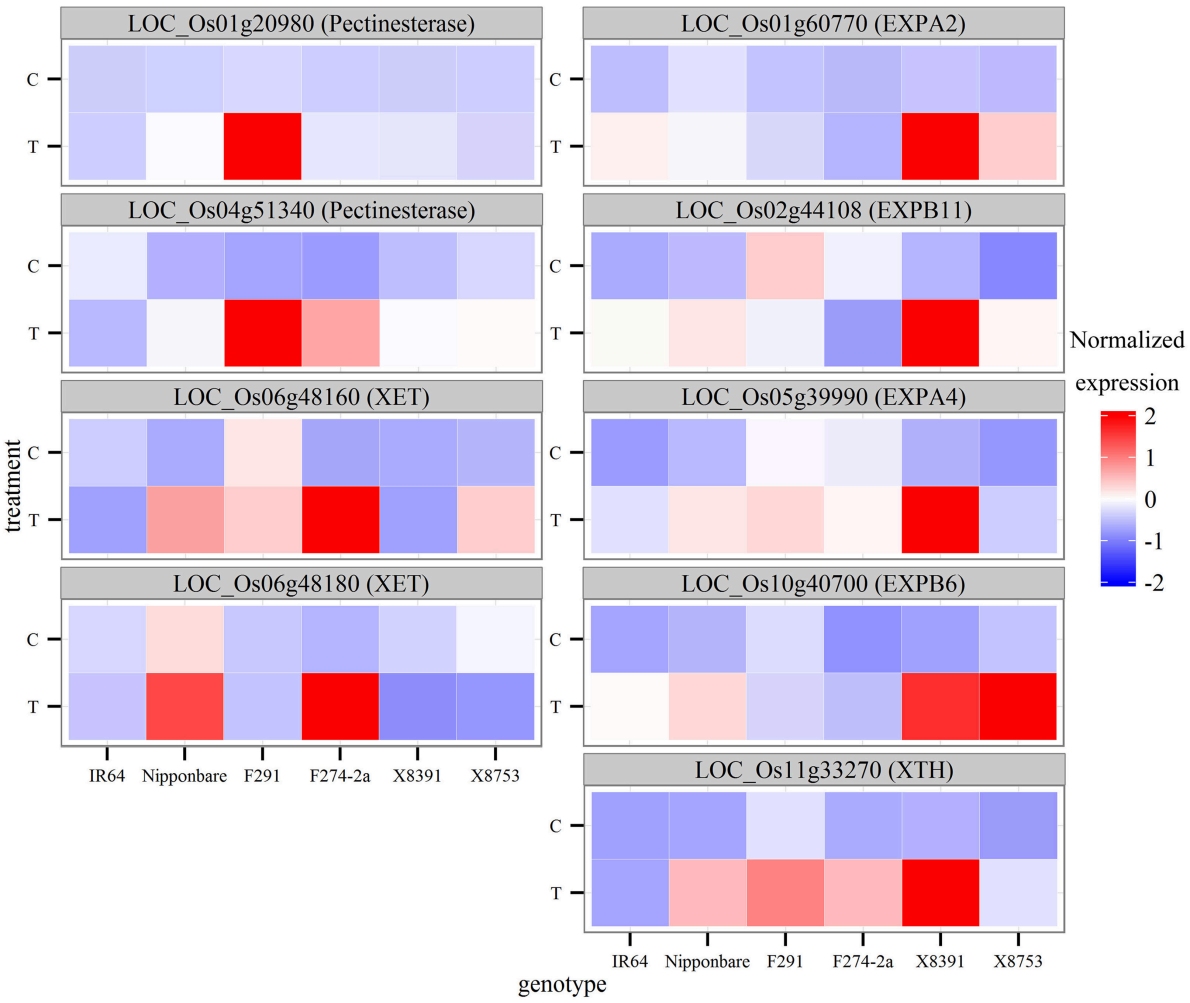
**Expression profiles of the nine cell-wall-related genes in the six rice genotypes**. The expression profiles of these nine genes were annotated with respect to cell wall biosynthesis and loosening. These profiles were then normalized for comparison across the six rice genotypes.

Cell elongation contributes significantly to coleoptile growth, and the genes encoding several cell wall loosening proteins, such as expansins, have been shown to be uniquely expressed under anoxia (Lasanthi-Kudahettige et al., 2007). Thus, to further investigate the contribution of cell wall-related genes to coleoptile elongation across our diverse genotypes, we examined the responses of over 100 genes involved in cell wall growth and loosening from the literature (Cosgrove, 2005) and from MapMan annotation bins. The results of this analysis show that 20 genes were differentially regulated (*P* < 0.05) by submergence in at least one genotype (Figure 6, Table S11). Interestingly, however, these results did not demonstrate a consistent expression pattern across diverse genotypes; for example, the gene for expansin B6 (*EXPB6, LOC_Os10g40700*) was significantly induced in the 8391 and 8753 genotypes but not in IR64 and the two RILs varieties, while the pectinesterase gene (*LOC_Os04g51340*) was only up-regulated in the two RILs. The results show that in the 8,391 genotype which exhibited the strongest coleoptile elongation significant induction of five cell wall-related genes was observed. At the same time, xyloglucan endotransglucosylase/hydrolase (XET) and pectinesterase genes were only slightly induced in this genotype (Figure 6).

In addition to genes related to the cell wall, those that respond to ethylene also play important roles in tolerance to submergence, including the ethylene response factor (ERF) genes *Sub1* and *SNORKEL* (Fukao et al., 2006; Xu et al., 2006; Fukao and Bailey-Serres, 2008a,b; Hattori et al., 2009). Because previous studies have demonstrated increased ethylene accumulation in young seedlings of the flood-tolerant rice variety “Khao Hlan On” in comparison to the sensitive IR42, this suggests that ethylene biosynthesis genes are involved in submergence tolerance during germination (Ismail et al., 2009). Indeed, of the candidate genes identified in this study, two ERF genes (*LOC_Os01g21120* and *LOC_Os07g47790*) were induced in all genotypes, although higher expression was seen in tolerant lines compared to the sensitive IR64 (Figure S6). Hence, because ethylene biosynthesis relies on the rapid oxidation of l-aminocyclopropane l-carboxylic acid (ACC), and because 22 genes that encode ACC oxidase (ACO) have been annotated in the rice genome, we investigated their expression patterns (Table S12). The results of this investigation show that one *ACO* gene (*LOC_Os01g39860*) was strongly induced in all genotypes when submerged, while another (*LOC_Os09g27820*) was expressed at higher levels in the five tolerant genotypes but was barely detectable in IR64.

In summary, although cell elongation and ethylene signaling pathways play important roles in the submergence response of rice seedlings, with some genes conservatively regulated in all genotypes, still more are regulated in a genotype-specific pattern, contributing to the higher tolerance of these lines. The results of this study illustrate the complexity of transcriptomic fine-tuning in response to submergence in rice seedlings from diverse genotypes.

## Conclusion

We present the first investigation of whole genome transcriptome profiles of diverse rice varieties that exhibit coleoptile growth variation when submerged. Our results highlight the genes that contribute to the essential mechanisms of submergence tolerance in rice, including carbohydrate metabolism, pyrophosphate-dependent energy conservation, and ethylene signaling pathways. We also show that the differential expression of genes between diverse genotypes has contributed to significant variation in submergence tolerance between tolerant and sensitive genotypes. Thus, in combination with the genotype-specific regulation observed in diverse rice varieties, our results suggest that coleoptile growth under water is fine-tuned at the transcriptional level, although how potential epistatic interactions and structural variations of candidate genes affect elongation rate remains to be investigated. This work highlights the importance of studying expression profiles across a diverse genetic background, as well as the potential for identifying favorable alleles for breeding tolerant rice varieties.

## Author contributions

SKH performed the experiments. SKH and CWT conceived and designed the experiments, analyzed the data and wrote the manuscript.

### Conflict of interest statement

The authors declare that the research was conducted in the absence of any commercial or financial relationships that could be construed as a potential conflict of interest.
