# Supplementary material for: RNA-Seq Analysis of Diverse Rice Genotypes to Identify the Genes Controlling Coleoptile Growth during Submerged Germination
Source: Front Plant Sci. 2017 May 15;8:762. doi: 10.3389/fpls.2017.00762 (PMC5430036; doi:10.3389/fpls.2017.00762)
Supplement: Supplementary file 4 [file Table4.DOCX]

**Table S4** Correlation table to show the differential expression of selected genes between RNA-seq and qRT-PCR.

| Selected genes | Description | Average log_2_ (T/C) ^a^ | | Correlation ^b^ |
| --- | --- | --- | --- | --- |
|  |  | RNA-Seq | qRT-PCR |  |
| LOC_Os01g11730 | Putative esterase | 3.85 | 1.08 | 0.75 |
| LOC_Os01g53930 | HXK6 | -1.11 | -1.41 | 0.76 |
| LOC_Os01g60770 | EXPA2 | 2.32 | 1.61 | 0.63 |
| LOC_Os01g67030 | Membrane protein-like | 7.59 | 1.48 | 0.78 |
| LOC_Os02g38920 | G3PDH | 4.05 | 4.03 | 0.83 |
| LOC_Os10g40510 | LTPL144 | 8.08 | 6.21 | 0.96 |
| LOC_Os11g10480 | ADH1 | 3.21 | 3.87 | 0.75 |
| LOC_Os11g10510 | ADH2 | 6.18 | 5.62 | 0.84 |
| Average |  |  |  | 0.79 |

a: log_2_-transformed fold change between the average expression of the submerged (T) and control (C) seedlings of six genotypes.

b: Pearson’s correlation coefficient evaluated for each gene based on log_2_-transformed relative expression level of 12 samples determined by RNA-seq and qRT-PCR.
