# Supplementary material for: RNA-Seq Analysis of Diverse Rice Genotypes to Identify the Genes Controlling Coleoptile Growth during Submerged Germination
Source: Front Plant Sci. 2017 May 15;8:762. doi: 10.3389/fpls.2017.00762 (PMC5430036; doi:10.3389/fpls.2017.00762)
Supplement: Supplementary file 14 [file Presentation4.PPTX]

## Slide 1
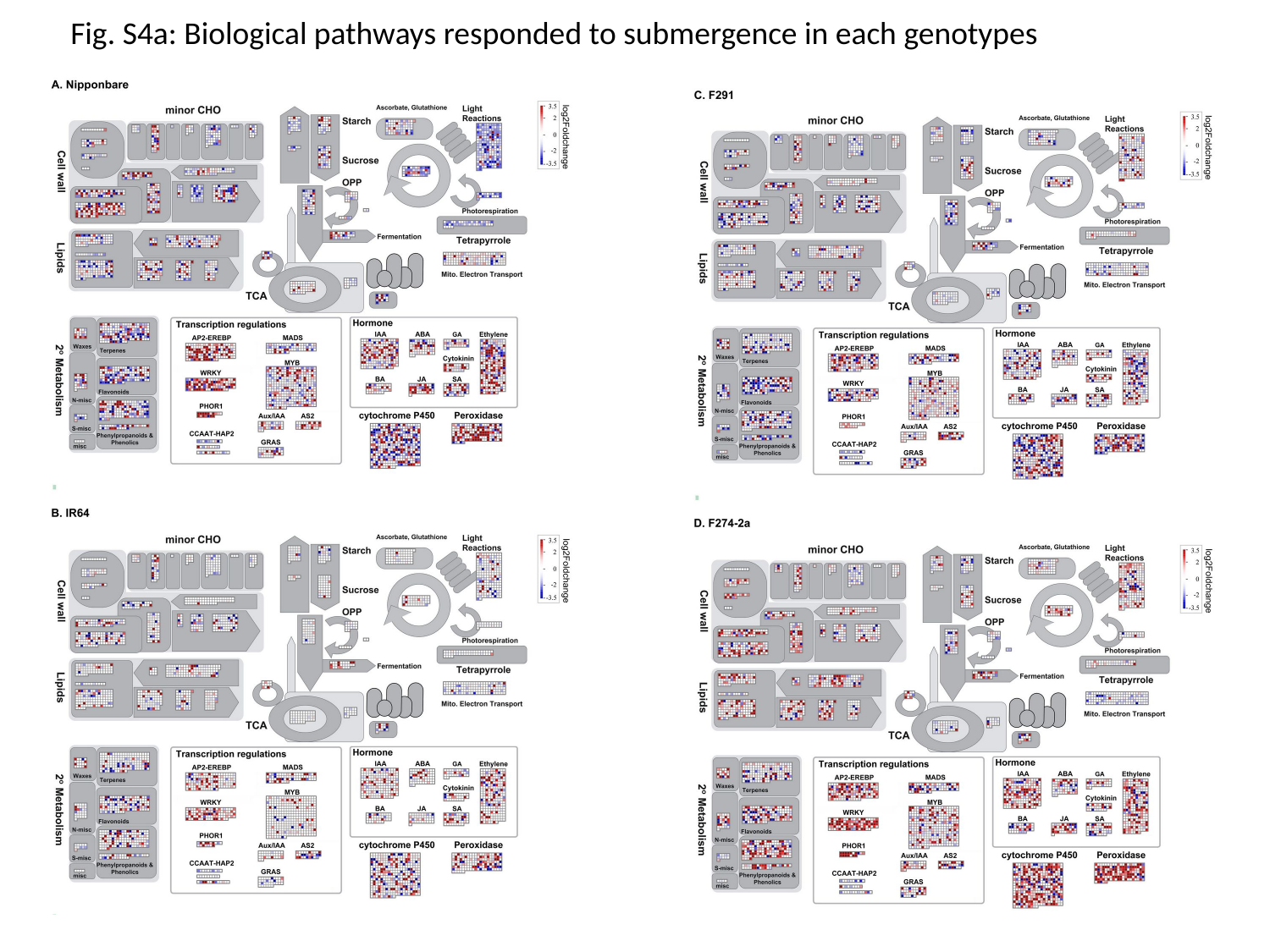

Fig. S4a: Biological pathways responded to submergence in each genotypes

## Slide 2
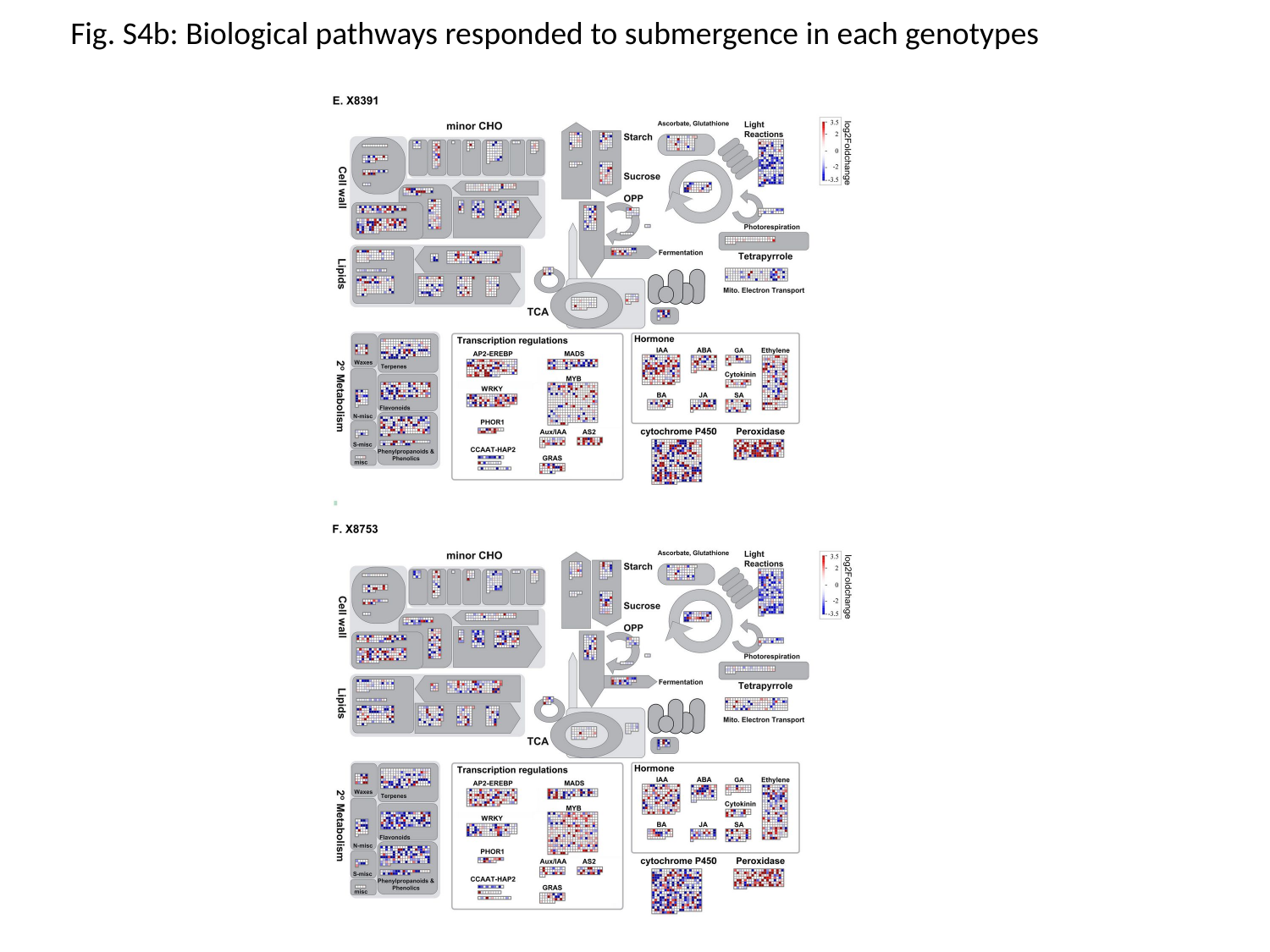

Fig. S4b: Biological pathways responded to submergence in each genotypes
