# Supplementary figures and images for: RNA-Seq Analysis of Diverse Rice Genotypes to Identify the Genes Controlling Coleoptile Growth during Submerged Germination
Source: Front Plant Sci. 2017 May 15;8:762. doi: 10.3389/fpls.2017.00762 (PMC5430036; doi:10.3389/fpls.2017.00762)

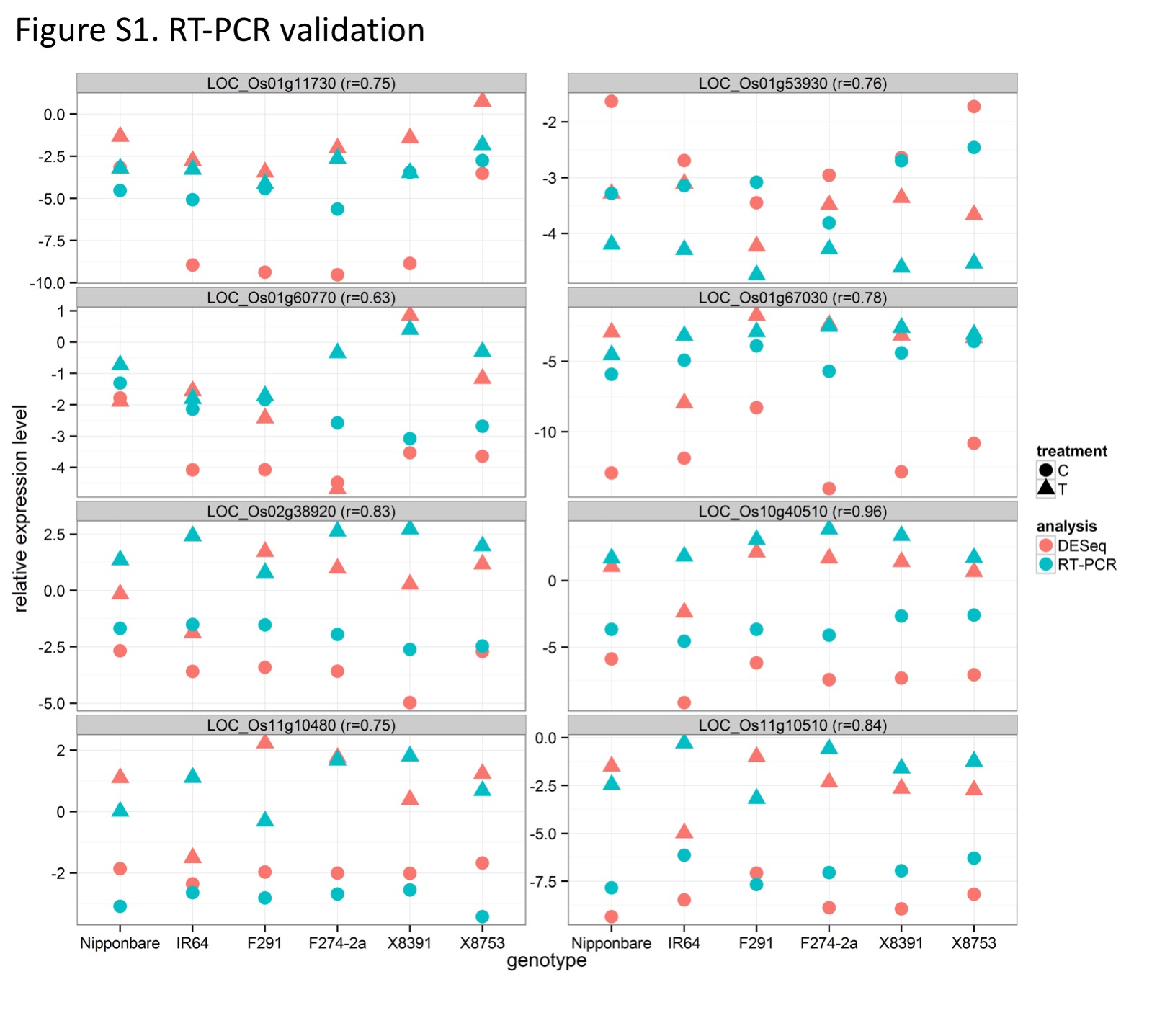

Supplement: Supplementary file 15 [file Image1.jpg]

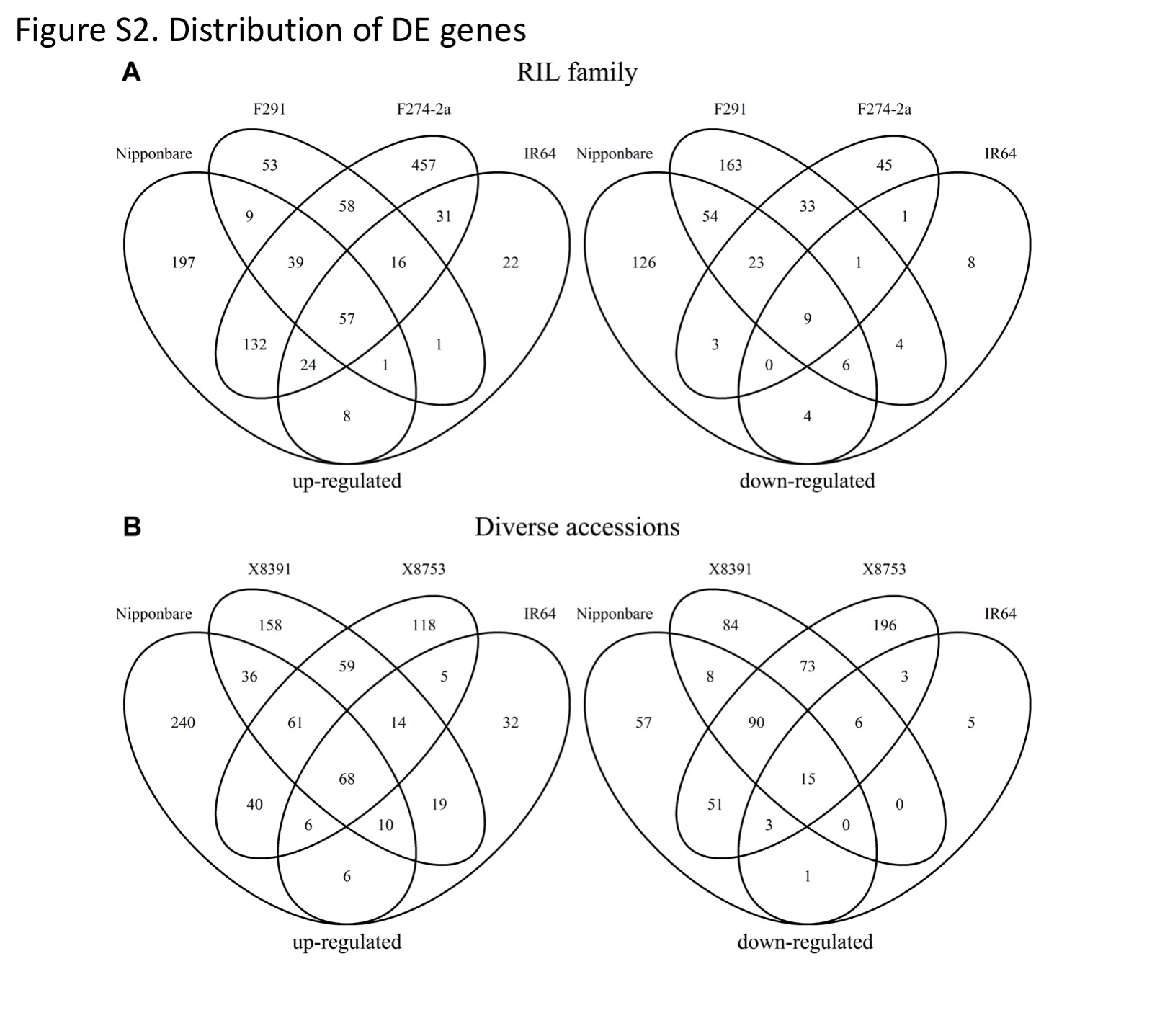

Supplement: Supplementary file 16 [file Image2.jpg]

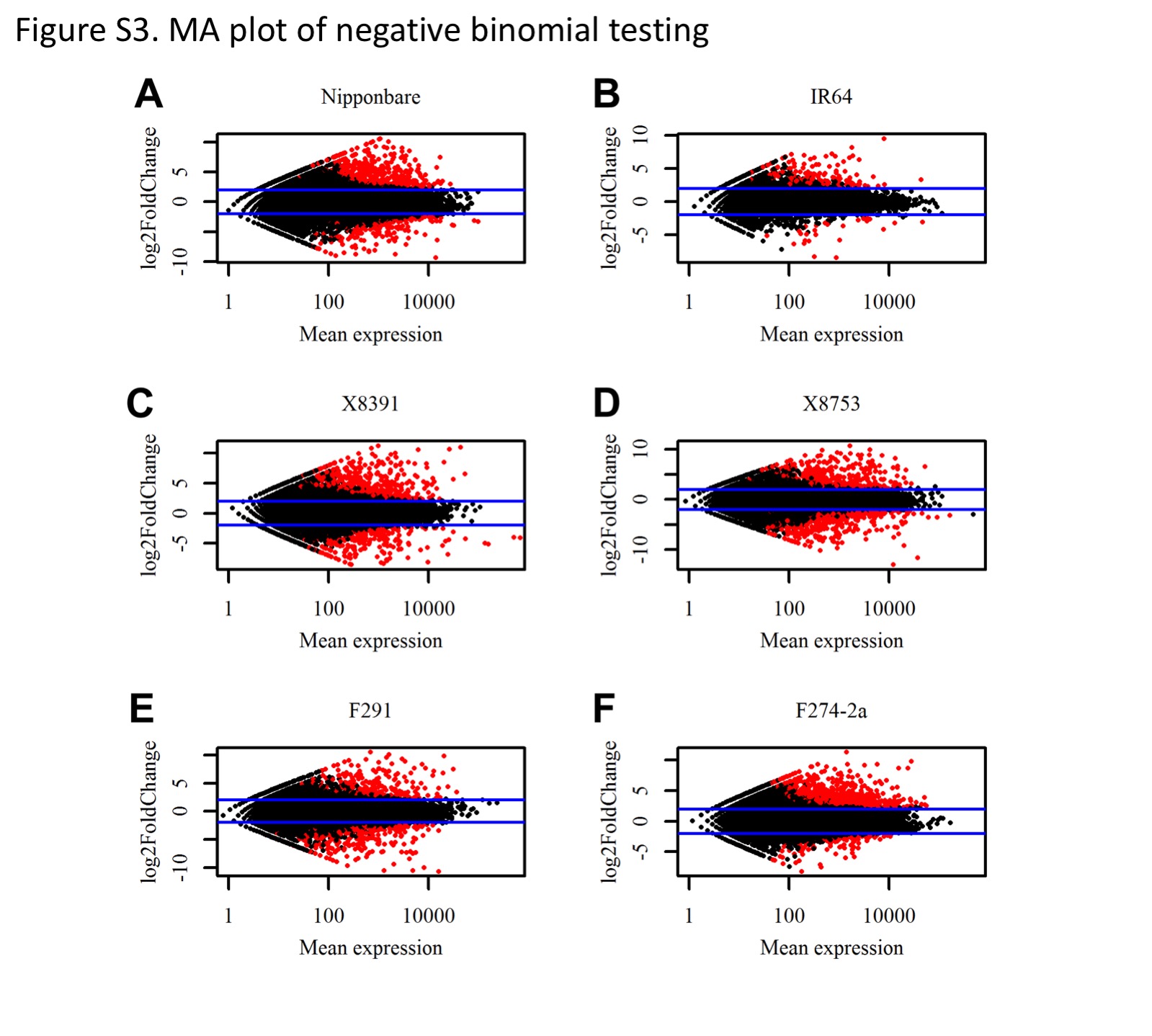

Supplement: Supplementary file 17 [file Image3.jpg]

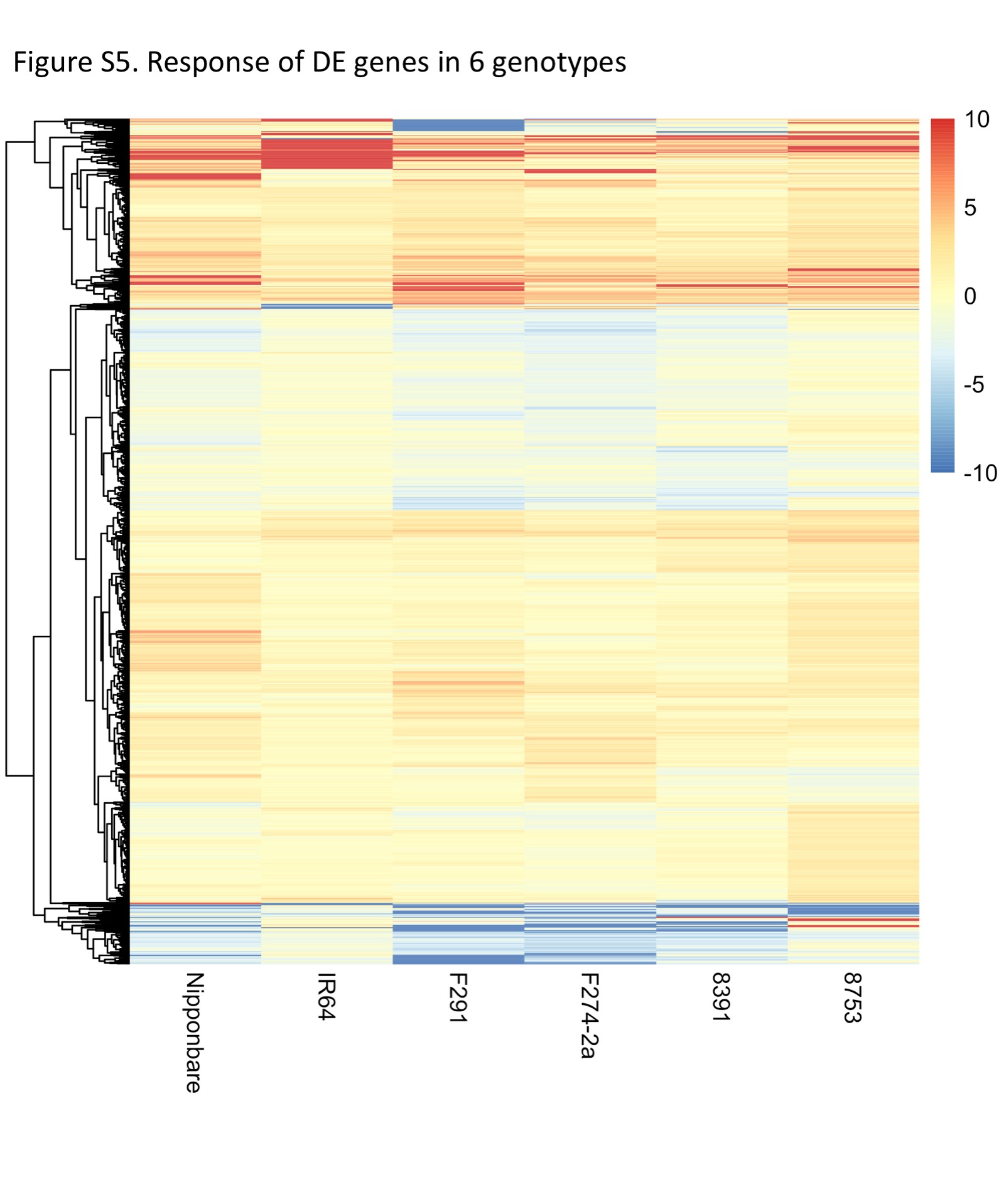

Supplement: Supplementary file 18 [file Image5.jpg]

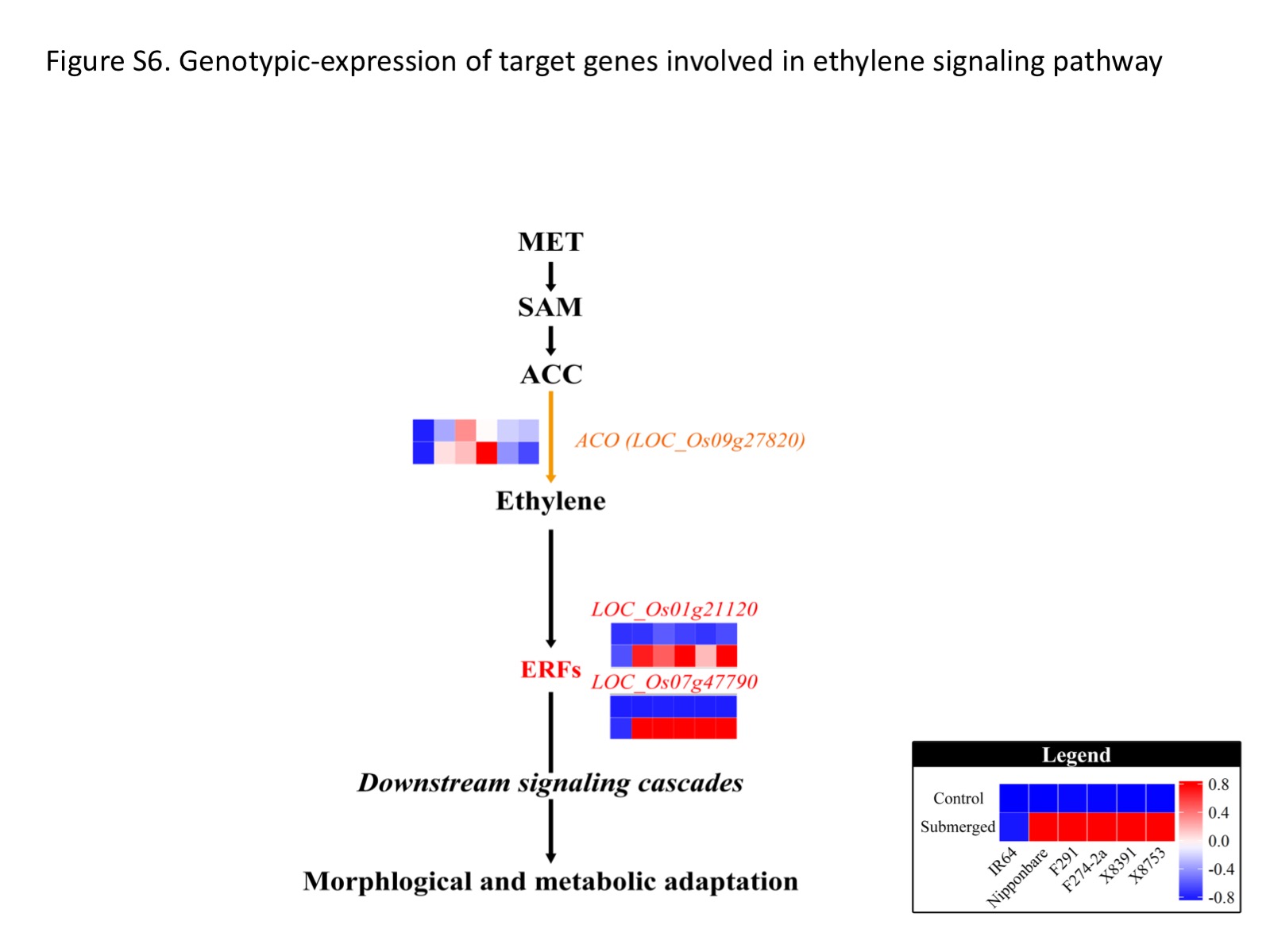

Supplement: Supplementary file 19 [file Image6.jpg]
